# Balneotherapy using thermal mineral water baths and dermatological diseases: a systematic review

**DOI:** 10.1007/s00484-024-02649-x

**Published:** 2024-03-26

**Authors:** Carmela Protano, Matteo Vitali, Andrea De Giorgi, Daniela Marotta, Serena Crucianelli, Mario Fontana

**Affiliations:** 1 Department of Public Health and Infectious Diseases, Sapienza University of Rome, P.Le Aldo Moro 5, 00185 Rome, Italy; 2 Department of Clinical Internal, Anesthesiological and Cardiovascular Sciences, Sapienza University of Rome, P.Le Aldo Moro 5, 00185 Rome, Italy; 3 Department of Biochemical Sciences, Sapienza University of Rome, P.Le Aldo Moro 5, 00185 Rome, Italy

**Keywords:** Balneotherapy, Dermatological diseases, Systematic review as topic, Mineral water

## Abstract

Balneotherapy includes practices and methods using medically and legally recognized mineral-medicinal waters, muds and natural gases from natural springs for therapeutic purposes. One of the most widely used method in balneotherapy is bathing with thermal mineral water. In the course of the years, scientific community has produced an increasing number of evidences that this practice is an effective method for treating signs and symptoms of several pathologies such as rheumatic, cardiovascular and dermatological diseases. This systematic review is aimed at evaluating the effect of balneotherapy with thermal water baths as a treatment to manage signs and symptoms of patients affected by all types of dermatological diseases. The systematic review was conducted according to the PRISMA Statement, and its protocol was registered on PROSPERO platform (CRD42022295913). The research was performed on the databases Pubmed, Scopus, Web of Science and Cochrane. We included clinical trials evaluating the effects of balneotherapy using thermal mineral water baths for managing dermatological diseases in humans, published in English and Italian language. Eight studies were included, seven of them enrolled adults affected by psoriasis and one studied atopic dermatitis patients. The common result of all the articles included was a clear improvement of signs and symptoms of psoriasis and eczematous diseases after use of thermal mineral water baths. These effects seem to be strictly related to physical and chemical properties of thermal water used for balneotherapy. However, studies in this field are still limited to support robust evidence of the effectiveness of balneotherapy using thermal mineral water baths and often their quality is low. Thus, new clinical studies need to be carried out, using more correct methods for conducting the studies and for processing statistical data.

## Introduction

Balneotherapy includes practices and methods using medically and legally recognized mineral-medicinal waters, muds and natural gases from natural springs for therapeutic purposes (Gálvez et al. 2018). One of the most widely used method in balneotherapy is bathing with thermal mineral water (Uzunoglu et al. 2017). This practice has proven positive effects on health depending on its physical and chemical-physical properties, such as temperature, composition and concentration of minerals contents, osmotic pressure and electrical conductivity (Fioravanti et al. 2011). For example, a high temperature of water can trigger anti-inflammatory and immunomodulating effects due to the release of mediators as β-endorphins, enkephalins and irisin (Borroni et al. 2013). Particularly, β-endorphins and enkephalins influence pain perception and regulate the proliferation of the immune cells (Nissen et al. 1998), whereas irisin improves metabolic and cognitive skills (Sacerdote et al. 2001). Besides, when the temperature of thermal water is high, it can act on dilatation of capillaries, increasing the blood flow and decreasing fibrinogen levels, finally improving the thrombotic profile of those undergoing balneotherapy (Aydin et al. 2013; Qiu et al. 2014). In addition, there are several positive outcomes related to the specific chemical contents (and their concentrations) of thermal water, such as sulfur, manganese, magnesium, selenium, strontium, silicon and bicarbonates (Lee et al. 2014; Rodrigues et al. 2017). Given these properties, in the course of the years, scientific community has focalized the attention on the positive effects of balneotherapy, producing an increasing number of evidences that balneotherapy is an effective method for managing pain (Antunes et al. 2019) and for treating signs and symptoms of several pathologies such as rheumatic (Bernetti et al. 2020), cardiovascular (Oyama et al. 2013) and dermatological diseases (Liang et al. 2015). In particular, dermatological diseases are one of the most common pathologies for which balneotherapy involving thermal mineral water baths is used (Huang et al. 2018). This is of great concern for public health because skin conditions are the most common cause for general practice consultation. Indeed, there are more than 3,000 skin diseases (both acute and chronic) affecting until to 70% of people worldwide and posing on patients a relevant burden in terms of quality of life loss and sanitary costs. The high prevalence of dermatological diseases together with their costs has to be carefully considered in planning dermatological care of patients (Richard et al. 2022), and effective treatments are needed in order to manage these diseases. Balneotherapy results an effective treatment for several skin conditions. For example, scientific evidence demonstrated a positive effect of this practice on psoriasis both in term of reducing collateral effects, as skin irritation and pruritus, as in term of safety, since it is a treatment not interfering with any metabolic comorbidities nor drugs, as typically occurs with pharmacological therapy (Timis et al. 2021). In this context, multiple studies reported as an effective balneotherapeutic treatment should last three to four weeks (Péter et al. 2017; Darlenski et al. 2021), although a significant improvement of the PASI index (Psoriasis Area Severity Index – severity score of psoriatic affected area) together with an improvement of life quality can be observed since the first session (Peroni et al. 2008). Besides, a recent narrative review evidenced a great positive effect of balneotherapy on psoriasis and atopic dermatitis and other dermatological conditions and diseases such as pruritus, prurigo, lichen ruber planus, acne vulgaris, and seborrheic dermatitis (Cacciapuoti et al. 2020). In addition, a recent systematic review demonstrated the potential positive effects of balneotherapy on dermatological diseases, but it was focalized on specific conditions such as psoriasis and atopic dermatitis (Moini Jazani et al. 2023). In our knowledge, no recent systematic review summarizes the scientific evidence on balneotherapy and all the known dermatological diseases. This systematic review is aimed at evaluating the effect of balneotherapy using thermal mineral water baths as a treatment to manage signs and symptoms of patients affected by all type of dermatological diseases.

## Materials and methods

### Research strategy

The present review has been performed following the PRISMA (Preferred Reporting Items for Systematic Reviews and Meta-Analyses) Statement (Page et al. 2021). The protocol of the review was registered on PROSPERO platform with the following ID: CRD42022295913.

We interrogated the following databases: PubMed (Medline), Scopus, Web of Science (Science and Social Science Citation Index) and Cochrane Library. The research was performed using the keywords and MeSH terms using Boolean operators as AND–OR utilizing the following string: “Balneotherapy” OR “thermal water” OR “mineral water” AND derm*. We choose these keywords and MeSH terms because we focalized the attention on the effect of balneotherapy using thermal mineral water baths. The research included all the articles published up to June 15th 2023.

### Inclusion and exclusion criteria

All the articles, regardless of the language, aimed at evaluating the effect of balneotherapy using thermal mineral water baths for the management of dermatological diseases in human beings were included in the present review. Any experimental study on humans was considered eligible, whereas case reports, case series, letters to editors, commentaries, editorials, reviews and observational studies were excluded. All the references cited, both in critical and systematic review and/or in meta-analysis, have been screened in order to detect further references worth mentioning. Any article not matching the inclusion criteria was excluded.

PICOS model was used for structuring the research question, as follows:

- Population: Patients having a dermatological disease.
- Intervention: Balneotherapy using thermal mineral water baths.
- Control: Bathing in drinking water or procedures other than balneotherapy.
- Outcomes: Improvement of one or more symptoms and signs of the studied dermatological disease.
- Study: experimental study on humans.

The exclusion criteria led to reject all reports not fulfilling both the requirements of this review and the predetermined inclusion criteria.

The references of the selected articles have been copied on Zotero citation management software (RRID:SCR_013784) to delete any duplicate and to evaluate the relevance of each article. Titles and abstracts of potentially eligible studies were independently screened by four investigators (M.V., A.D.G., D.M., C.P.). The group of the four investigators included two content experts (M.V. and C.P.) that assisted with the screening and reviewing process and two graduate students (A.D.G. and D.M.) that assisted with searching, screening, evaluating process. Hereafter, the four researchers focused independently on the full version of the potentially relevant articles. Any disagreement about the selection has been discussed and resolved. In particular, the same four investigators discussed each conflict until agreement was reached or asked to the subject matter expert of the team (M.F.) to resolve the conflict.

### Quality evaluation of single study

The eligible full texts resulting at the end of the review process were almost all controlled clinical trials with the exception of one, concerned a not controlled clinical study (open, with no control group) (Costantino and Filippelli 2014).

To date, there is not a universally accepted checklist to assess the methodological quality of clinical studies dealing with any other therapeutic treatments but pharmacological one. Following the methodology previously used by other systematic reviews in this field (Santos et al. 2016), evaluating not pharmacological treatment (Alkaduhimi et al. 2018; Kamioka et al. 2011; Liu et al. 2020), we assessed the quality of any single study using the CLEAR NPT checklist (Checklist to Evaluate a Report of a Non pharmacological Trial). This checklist was specifically thought to evaluate not pharmacological clinical trials by a group of experts, using the Delphi method. It consists of 10 questions having dichotomous answers (yes/not) plus a “not reported” option. In particular, the questions of the checklist investigated the standardization of the intervention, care provider influence, and other measures for minimizing the potential bias deriving from lack of blinding of participants, care providers, and outcome assessors (Boutron et al. 2005). Moreover, we assessed the statistical validity and the quality of adverse /collateral events evaluation using the checklist used by Forestier et al (2016).

Four investigators (M.V., A.D.G., D.M., C.P.) independently evaluated the risk of bias for each single study and the quality of any article included in the review using the above-described checklists. Any incoming disagreement was discussed and resolved. In particular, the same four investigators discussed each conflict until agreement was reached or asked to the subject matter expert of the team (M.F.) to resolve the conflict. According to what pre-established by the authors of both checklists (Forestier et al. 2016): a score ranging from 10 to 8 corresponds to a low risk of bias, a score ranging from 7 to 5 represents a moderate risk and a score lower than 5 stands for a high risk of bias.

Regarding the uncontrolled clinical study (open, no control group), comparing the effects of balneotherapy using thermal mineral water baths on a group of patients before and after treatment, the quality assessment and the evaluation of the bias risk were performed considering the scientific value of the journal, the sample size and the methods used to evaluate patients, in accordance to previous systematic review in this field (Fraioli et al. 2018). However, due to the lack of a control group, the risk of bias has been considered higher.

## Results

**Fig. 1:**
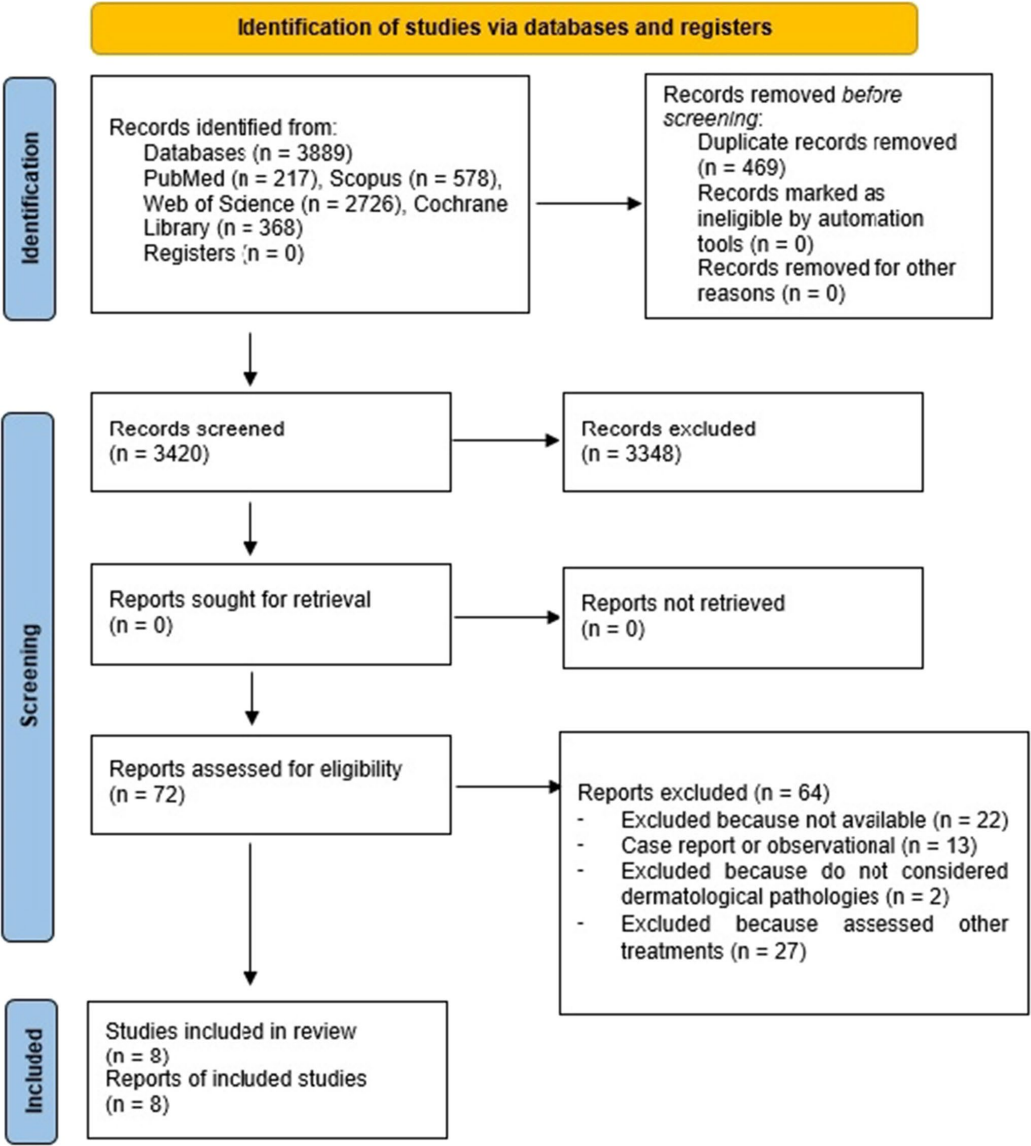
Flow chart describing the research strategy

Figure 1 reports the details of the review process.

Eight studies have been finally included in the review and undergone to the quality synthesis. The initial research performed on the databases Pubmed, Scopus, Web of Science and Cochrane produced 3,889 bibliographic citations; 3,420 left after the duplicates removal, of which 3,348 were further excluded because titles and abstracts not responded to the eligibility criteria. 22 articles resulted not available, 42 were excluded because they reported observational studies or not evaluating dermatological diseases or because coupling balneotherapy using thermal mineral water baths to other treatments (UV radiations, Dead Sea salts, topic application or parenteral consumption of thermal water). The references of the selected articles were further assessed for any other relevant citations, but no articles met the inclusion criteria.

Data related to each included article are summarized on Table 1. In particular, we reported the bibliographic references, the thermal centre, the country in which the study was set, the source of funding, the study design, the type of dermatological disease, features of the population studied in terms of gender and age, description of the balneotherapy intervention, description of the control intervention, main results, authors’ conclusions, risk of bias.

**Table 1:** Features of the studies included in the review

Study Country Study design Source of funding Dermatological diseases	Sample Size and sample features	Balneotherapeutic treatment	Interventions on other groups/controls	Original Authors’ results and conclusions	Quality evaluation according to CLEAR NTP checklist
Even-Paz et al. 1996 Israel Controlled clinical study Not reported Psoriasis	81 (47 cases, 34 controls); age ranging from 16 e 80 years	Four consecutive weeks of bathing in Dead Sea water or bathing + sun exposition	Just sun light exposure	The PASI significantly improved at the end of all the three tested treatments	High risk of bias
Zumiani et al. 2000 Italy Randomized controlled clinical study Not reported Eczematous dermatitis (atopic and contact dermatitis)	48 (25 cases, 23 controls); 23 M e 25F mean age equal to 24,5 years	20 min/day of bathing in warm thermal water (37 °C) for 12 times within 20 days since treatment beginning	20 min/day of bathing in warm potable water (37 °C) for 12 times within 20 days since treatment beginning	Positive improvement of the clinical index in both groups, with a significant improvement for patients treated with thermal water than those treated with potable water. Skin moisture rapidly increased after bathing in thermal water	Moderate risk of bias
Léauté-Labrèze et al. 2001 France Randomized controlled clinical study Not reported Psoriasis	71 (22 cases with thermal waters, 21 cases with thermal water and UVB, 24 cases with UVB, 4 “dropped out”); 46 M e 25 F; age ranging from 19 e 76 years (mean 49 years)	20 min/day of immersion in warm thermal water (35 °C-37°C) for five days a week for three consecutive weeks + intervention group bathing in thermal water + phototherapy with UVB rays	Phototherapy with UVB rays only	A certain improvement of the PASI was described at the end of the three tested treatments and after one year	Moderate risk of bias
Brockow et al. 2007 Germany Single blind controlled clinical study Not reported Psoriasis	143; 53 F e 90 M; mean age equal to 49,9 ± 13,3	20 min/day of immersion in thermal water for three days up to symptoms remission or at maximum for six weeks (18 sessions), each session followed by UVB phototherapy	Use of phototherapy only with UVB, once a day for three days/week up to symptomatology remission or maximum up to six weeks (18 sessions)	Significant improvement of the PASI at the end of both tested treatments Higher reduction of dermatological symptomatology for the combination balneotherapy + phototherapy than for phototherapy only	Low risk of bias
Battaglia et al. 2008 Italy Not randomized controlled clinical study Not reported Psoriasis	100 (50 cases with balneotherapy + phototherapy, 50 cases with balneotherapy only); 44 F e 56 M; age ranging from 21 to 75 years	20 min/day of immersion in warm thermal water (36 °C) resting the further 15 min, once a day for 12 days + phototherapy with UVB narrow band with increasing dosage	20 min/day of bathing in warm thermal water (36 °C) resting the further 15 min, once a day for 12 days	Significant improvement of psoriatic symptomatology, with erythema, desquamation, and hyperkeratosis reduction at the end of the treatments. Significant improvement of the PASI at the end of both tested treatments	High risk of bias
Peroni et al. 2008 Italy Not randomized controlled clinical study Private Psoriasis	280 (156 cases with balneotherapy only, 124 cases with balneotherapy + phototherapy); age ranging from 18 to 85 years	20 min/day of bathing in warm thermal water (36°-37 °C) once or twice a day on a group of 77 patients for one week and a group of 79 for two weeks	20 min, once -twice a day, of bathing in warm thermal water (36–37 °C) followed by 15 min of UVB irradiation with increasing intensity, a group of 40 patients for one week and a group of 84 for two weeks	Significant improvement of the PASI at the end of both the treatments tested, after one and two weeks. The improvement of the results has been confirmed by the SAPASI and by the questionnaire on life quality Skindex-29	Moderate risk of bias
Morri et al. 2012 Italy Randomized controlled clinical study Not reported Psoriasis	60 (divided into 3 groups of 20); age ranging from 18 to 65 years	20 min/day of bathing in thermal water, followed by 15 min of rest using cotton towels + a group having balneotherapy and phototherapy using UVB rays, twice a week for 24 times	24 applications, twice a week of phototherapy only with UVB rays	The PASI resulted significantly improved at the end of tested treatments	Moderate risk of bias
Costantino and Filippelli 2014 Italy Uncontrolled clinical study Not reported Psoriasis	35; 23% M, 77% F; mean age: 56 ± 19 years (min–max: 17–85 years)	15 min/day of bathing in warm thermal water (37 °C) 12 times, followed by 15–20 min of controlled, once a day for six days resting the seventh	Not performed	Significant reduction of pruriginous symptomatology, improvement of the NRS score and PASI, improvement of quality of life, improvement of the anxiety-depressive symptomatology related to the dermatological disease	High risk of bias

PASI index = Psoriasis Area Severity Index; SAPASI index = Self-Administered Psoriasis Area and Severity Index; NRS = Numerical Rating Scale

All the eight studies included in the review were clinical trial, seven of them were controlled (Battaglia et al. 2008; Brockow et al. 2007; Even-Paz et al. 1996; Léauté-Labrèze et al. 2001; Morri et al. 2012; Peroni et al. 2008; Zumiani et al. 2000), while one was open, without a control group (Costantino and Filippelli 2014). Almost all the studies enrolled adults affected by psoriasis and one (Zumiani et al. 2000) involved patients affected by eczematous dermatitis (atopic or contact dermatitis). In this case, balneotherapy were used as an intervention characterized by 20–30 min of bath, once or twice a day for 2–3 weeks’ duration, coupled or not with Sun exposure or UV radiations both for cases and controls (otherwise, the studies have been rejected). The PASI index (Psoriasis Area Severity Index) is considered the outcome index in most of the studies. Some studies considering even the VAS index (Visual Analogue Scale), the NRS index (Numerical Rating Scale), several dermatological symptoms, clinical objective evaluation of patient’s subjective one, skin moisture, quality of life. Some outcomes have been evaluated just at the end of the treatment and/or at the end of up to 3 months’ follow-up period, and compared to the pre-treatment situations or to a control group.

According to the checklist CLEAR NTP or Forestier et al. (2016) for the risk of bias and quality of the studies, three studies have a high risk of bias (included the uncontrolled one), four have a moderate risk and one has a low risk. Moreover, some improvable methodological aspects been underlined by quality, statistical and the significance evaluation. The agreement expressed by the outcomes reached by all the studies allow to lower the bias burden while strengthening the scientific evidences. All the studies showed a significant improvement of one or more signs and/or peculiar symptoms of psoriasis and eczematous dermatitis. Psoriasis is probably the most investigated dermatological disease; seven studies of the review report an improvement of patient’s status after balneotherapy expressed in term of:

1. PASI index improvement (7/7 articles);
2. reduction of dermatological symptoms (2/7);
3. SAPASI index (self-administered psoriasis area and severity index) improvement (1/7);
4. VAS index (Visual Analogue Scale) improvement (1/7);
5. improved quality of life (2/7).

Eczematous dermatitis, as demonstrated by studies focusing on this disease, showed some positive variation of clinical indexes when treated with thermal water rather than with potable water. Moreover, skin moisture rapidly improved after thermal bathing.

## Discussion

The main finding of the present systematic review is that all the articles included demonstrated a clear improvement of signs and symptoms of psoriasis and eczematous diseases after the treatment with balneotherapy using thermal mineral water baths. According to the authors of the considered articles and to the scientific literature in this field, thermal bathing triggers its clinical effects through a double mechanism: the former, non-specific (hydrotherapy) due to mechanical influences and hydrostatic pressure (crenotherapy) linked to physical and chemical properties of thermal water (Fioravanti et al. 2011; Forestier et al. 2016; Fraioli et al. 2018; O’Hare et al. 1985). As regards to crenotherapy, immersion in thermal waters, particularly in saline (sodium-chloride rich waters) and sulphurous waters, is widely considered an integrative and complementary dermatological treatment due to its keratolytic, regenerative and anti-oxidative effects (Soroka et al. 2008; Peinemann et al. 2020). Besides, several studies reported that chemicals such as sulfur, manganese, magnesium, selenium, strontium, silicon and bicarbonates in thermal water has immunomodulatory effect on skin disorders, especially on atopic dermatitis, contact dermatitis and psoriasis (Lee et al. 2014; Rodrigues et al. 2017). For example, the sulphur can inhibit T-cells proliferation and the production of interleukin -2, interleukin -8, interleukin -23, interleukin -17 and interferon-γ, finally improving inflammation related to the skin disorders previously mentioned (Mirandola et al. 2011). Besides, sulfur and magnesium have bactericide effect against *Staphylococcus aureus*, which is a typical strain colonizing and complicating skin lesions of atopic dermatitis (Akiyama et al. 2000; Scala et al. 2019), by reinforcing the skin barrier and the immune system, speeding up skin regeneration. An in vitro study demonstrated as thermal water rich in selenium and strontium when in contact with epidermidis cells, can inhibit the production of pro-inflammatory cytokines, particularly of interleukin 6 (Wollenberg et al. 1992). In a systematic review by Khalilzadeh et al. (2019) on the therapeutic effects of thermal water, has been observed as saline waters (sodium-chloride rich) presenting a high content of minerals, can reduce the enzyme leukocyte esterase, involved in psoriasis, the transforming growth factor-β (TGF-β), increased in psoriatic patients, the Langerhans cells of the epidermis and skin infections trough the removal of bacteria that contribute to seborrheic dermatitis and other dermatological diseases.

The present systematic review has some limitations. First of all, statistical analysis of the results of the studies included in the review was not performed because we considered all the dermatological diseases and, thus, it is not possible to compare changes in symptoms and signs of different pathologies. Besides, each included studies used different approaches both for conducting the trial and for evaluating changes in clinical symptoms and signs of the dermatological disease studied. However, this systematic review is the first giving a picture of the positive effects of balneotherapy using thermal mineral water baths on symptoms and signs of all type of dermatological diseases. Secondly, the quality of the studies resulted overall low, with three studies have a high risk of bias, four have a moderate risk and one has a low risk. Nevertheless, the findings of the studies included agree and evidenced a significant improvement of one or more specific signs and/or symptoms of the studied dermatological diseases after the balneotherapy treatments.

## Conclusion

The results of the present systematic review evidences that all the included studies demonstrated a significant improvement in signs and/or symptoms of the studied dermatological diseases. However, the quality of the studies included is not so high in several cases because the methodological approaches used to perform the study and/or the elaboration of the results were not completely appropriate. Therefore, it is necessary to carry out further clinical trials with more proper methods to conduct this kind of studies and more advanced techniques to perform statistical elaboration of data.

## Data Availability

The data that support the findings of this study are available from the corresponding author upon reasonable request.
